# Identification of Nonviable Genes Affecting Touch Sensitivity in *Caenorhabditis elegans* Using Neuronally Enhanced Feeding RNA Interference

**DOI:** 10.1534/g3.114.015776

**Published:** 2015-01-09

**Authors:** Xiaoyin Chen, Margarete Diaz Cuadros, Martin Chalfie

**Affiliations:** Department of Biological Sciences, Columbia University, New York, New York 10027

**Keywords:** *Caenorhabditis elegans*, mechanosensation, lethal, *mca-3*, *txdc-9*

## Abstract

*Caenorhabditis elegans* senses gentle touch along the body via six touch receptor neurons. Although genetic screens and microarray analyses have identified several genes needed for touch sensitivity, these methods miss pleiotropic genes that are essential for the viability, movement, or fertility of the animals. We used neuronally enhanced feeding RNA interference to screen genes that cause lethality or paralysis when mutated, and we identified 61 such genes affecting touch sensitivity, including five positive controls. We confirmed 18 genes by using available alleles, and further studied one of them, *tag-170*, now renamed *txdc-9*. *txdc-9* preferentially affects anterior touch response but is needed for tubulin acetylation and microtubule formation in both the anterior and posterior touch receptor neurons. Our results indicate that neuronally enhanced feeding RNA interference screens complement traditional mutageneses by identifying additional nonviable genes needed for specific neuronal functions.

The *Caenorhabditis elegans* genome contains 20,517 genes (WS245; WormBase 2014). Feeding RNA interference (RNAi) against ~86% of these genes, however, only identified 1722 genes whose reduction in expression produced a phenotype (Kamath *et al.* 2003). Of these 1722 genes, 1170 produced nonviable phenotypes. Although the low incidence of genes mutating to an obvious phenotype can be partially explained by ineffective systemic RNAi in the *C. elegans* nervous system and redundancy (Kamath *et al.* 2001), the high number of genes causing lethality when knocked down by RNAi suggests that genes needed for viability make up a considerable portion of the genome.

In *C. elegans* gentle touch along the body is sensed by six touch receptor neurons (TRNs; Chalfie and Sulston 1981; Chalfie and Au 1989). Saturated mutageneses for touch-insensitive mutants have identified 18 genes required for mechanosensation, including genes encoding components of the mechanotransduction complex, the cytoskeleton, the extracellular matrix, and genes affecting the development of the TRNs (Chalfie and Au 1989; Du and Chalfie 2001; O’Hagan and Chalfie 2006; Bounoutas and Chalfie 2007). None of these genes is essential. DNA microarrays identified additional genes that were highly expressed in the TRNs and that were also needed for TRN function or differentiation (Zhang *et al.* 2002; Zhang and Chalfie 2002; Topalidou and Chalfie 2011; Topalidou *et al.* 2011). These analyses, however, were biased against genes that act pleiotropically. Genes needed for touch sensitivity, but also for movement, fertility, or viability in other cells, would not have been identified easily.

In this paper we describe an RNAi-based screen to identify pleotropic genes needed for touch sensitivity. Because the nervous system is mostly dispensable for viability in *C. elegans*, neuronally enhanced feeding RNAi allows the screening of genes with nonviable phenotypes for neuronal phenotypes. We previously used neuronally enhanced feeding RNAi to identify several focal adhesion genes needed for optimal touch sensitivity (Calixto *et al.* 2010). The complete loss-of-function alleles of these genes cause lethality, but the touch insensitivity was confirmed in mosaic animals (Chen and Chalfie 2014). Here we take advantage of a previous genome-wide screen for genes that cause lethality and developmental arrest when mutated (Fraser *et al.* 2000; Kamath *et al.* 2003) to identify genes that are involved in TRN function and development. We found 61 such genes (five of which had been previously identified) and confirmed 18 of the new genes using partial loss-of-function alleles or maternally rescued null alleles.

## Materials and Methods

### Strains

*C. elegans* strains were maintained at 15° as described by Brenner (1974). EG3350[*oxEx593(mca-3p*::*gfp)*] (Bednarek *et al.* 2007) was a gift from Eric Jorgenson; CB47 [*unc-11(e47) I*], GS2526 [*arIs37 I*; *mca-3(ar492) dpy-20(e1282) IV*], RB1887 [*tom-1(ok2437) I*], VC206 (*fzy-1&cyp-44A1(ok312) unc-4(e120)/mIn1 [mIs14dpy-10(e128)] II*), VC3455 (*+/hT2 [bli-4(e937) let-?(q782) qIs48] I*; *kin-18(ok395)/hT2 III*), VC556 {*+/hT2 [bli-4(e937) let-?(q782) qIs48] I*; *txdc-9vps-16(ok776)/hT2 III*} (note: this paper renames *tag-170* as *txdc-9)*, SSM42 [*let-92(ok1537)/nT1qIs51 IV*; *+/nT1 V*], CB408 [*unc-43(e408) IV*], KY46 [*cab-1(tg46) X*], MT7553 [*dpy-19(e1259) sqv-3(n2842)/eT1 III*; *+/eT1 V*], VC724 (*mog-5(ok1101)/mIn1 [mIs14dpy-10(e128)] II*), PK172 (*ptc-1(ok122) unc-4(e120)/mnC1dpy-10(e128) unc-52(e444) II*), MT8626 [*goa-1(n3055) I*], VC1148 (*vha-5(ok1588)/nT1 [qIs51] IV*; *+/nT1 V*), VC927 (*+/hT2 [bli-4(e937) let-?(q782) qIs48] I*; *pnk-1(ok1435) I/hT2 III*), VC2226 (*cgt-3(ok2877)/mIn1 [mIs14dpy-10(e128)] II*), RB1278 (*let-502(ok1283)/hT2 [bli-4(e937) let-?(q782) qIs48] I*; *+/hT2 III*}, and VC1400 (*cdk-1(ok1882)/ hT2 [bli-4(e937) let-?(q782) qIs48] I*; *+/hT2 III*) were from the Caenorhabditis Genetics Center; TU3595 (Calixto *et al.* 2010), and TU4706 [*arIs37 I*; *mca-3(ar492)dpy-20(e1282) IV*; *uEx924(mec-17p*::*rfp*, *mec-3p*::*mca-3c)*], TU4707 (*+/hT2 [bli-4(e937) let-?(q782) qIs48] I*; *txdc-9vps-16(ok776)/hT2 III*; *uEx925[mec-17p*::*rfp*, *mec-3p*::*txdc-9]*) were from laboratory stocks. TU4707 and TU4706 were made by injecting VC556 and GS2526 with the indicated constructs, respectively.

### Constructs

The rescuing constructs of *mec-3p*::*txdc-9* and *mec-3p*::*mca-3c* were made using the Multisite Gateway Three-Fragment Vector system (Life technologies, Grand Island, NY). The plasmid *mec-17p*::*rfp* and the entry clones containing *mec-3p* and *unc-54 3′utr* were described by Chen and Chalfie (2015). *txdc-9* genomic sequence was amplified using the primers GGGGACAAGTTTGTACAAAAAAGCAGGCTTAatggccgctaatattcaacagcagt and GGGGACCACTTTGTACAAGAAAGCTGGGTGctaccaatcctcttcgttatcgtactcc. *mca-3c* cDNA sequence was amplified using primers GGGGACAAGTTTGTACAAAAAAGCAGGCTTAatgcccgaatatggtgcatcactg and GGGGACCACTTTGTACAAGAAAGCTGGGTGtagattgttcgtctccttgatatttccacg. These fragments were then inserted into pDONR221 using standard Gateway BP reactions to create the entry clones.

### Feeding RNAi screen

The list of lethal RNAi was pulled from WormBase WS200 (http://ws200.wormbase.org/) using WormMart (http://caprica.caltech.edu:9002/biomart/martview/eb8ae77c42524bf40ecbee9f5c7feab6) using the following phenotype codes: lethal, adult_lethal, embryonic_lethal, embryonic_terminal_arrest_variable_emb, embryonic_lethal_late_emb, larval_lethal, larval_arrest, late_larval_lethal, late_larval_arrest, paralyzed, one_cell_arrest_early_emb, and embryonic_terminal_arrest_variable_emb. The search was restricted to RNAi experiments described by Fraser *et al.* (2000) and Kamath *et al.* (2003), which used the Ahringer RNAi library.

Feeding RNAi treatments were performed as described by Calixto *et al.* (2010) in TU3595 for the screen. Animals were grown on six-well plates. Each RNAi bacterial strain was tested five times independently, and genes that produced touch insensitivity in at least four of five tests were considered to cause touch insensitivity. Positive RNAi constructs were sequenced to confirm their identities.

During the initial round of screen, 190 of the 849 RNAi constructs produced anterior touch insensitivity in our hands. Because the number of false-positive results is always equal or smaller than the number of all positives, our screen has an estimated maximum false-positive rate of ~20% (assuming that all 190 positives are false-positive). To reduce false-positive results, we performed multiple independent tests and scored an RNAi construct positive only if it produced touch insensitivity four of five times. Because the false-positive rate must be equal to or less than the total positive rate of 20%, the theoretical upper limit for the false-positive rate for five tests is C51×0.24×0.8+0.25=0.67%, corresponding to 0.67%×849=6 false positives of the 61 genes obtained from the screen. The actual false-positive rate, however, is likely to be much lower than this upper bound, given that a large fraction of the 190 positives from the initial round of the screen were real positives. Using the number of positives after five rounds of screens, we estimate that the real false-positive rate of a single round of screen to be [190−(61−6)]/849=16%. Using this estimate, we find the actual false-positive rate for five tests is C51×0.164×0.84+0.165=0.29%, corresponding to 0.29%×849=2 false-positive results.

### Touch sensitivity assay

We tested touch sensitivity as described by Chen and Chalfie (2014). Each animal was tested five times anteriorly and five times posteriorly (Hobert *et al.* 1999), or five times anteriorly only for RNAi tests, because feeding RNAi is less effective for posterior touch sensitivity (Calixto *et al.* 2010). A total of 10−20 animals were tested each time. The average and SEM of the response of at least three biological replicates were reported and used for statistical analyses.

### Microscopy

Animals were immobilized on 4% agarose pads with 30 mM sodium azide and imaged on a Zeiss Observer Z1 inverted microscope using a Zeiss Apochromat 40× 0.95 air objective for TRN antibody staining against MEC-7 and MEC-18, a Zeiss Apochromat 100× 1.4 oil objective for antibody staining against acetylated tubulin and MEC-2, and a Zeiss Neofluor 10× 0.3 objective for green fluorescent protein (GFP) imaging. The images were acquired with a Photometrics Coolsnap HQ2 camera using Zeiss Colibri 2 as the excitation source.

### Single-molecule messenger RNA (mRNA) fluorescent *in situ* hybridization (FISH)

Single-molecule mRNA FISH was performed as previously described (Topalidou *et al.* 2011).

### Antibody staining

Adult *txdc-9* and wild-type animals were fixed as described by Miller and Shakes (1995) using a fixation buffer containing 1% formaldehyde and 50% methanol. Other strains were fixed using a fixation buffer containing 1% paraformaldehyde and 20% methanol. Mouse anti-MEC-18 (1:200; S. Zhang and M. Chalfie, personal communication), mouse anti-MEC-2 (1:200; Zhang *et al.* 2004), mouse anti-MEC-7 (1:200; Hamelin *et al.* 1992) and rabbit anti-acetylated tubulin (1:200; 6-11B1, Life Technologies, Grand Island, NY), monoclonal primary antibodies were used for staining overnight at 4°. Alexa Fluor 488-conjugated goat anti-mouse (1:1000; Life Technologies) and Cy-3 goat anti-rabbit (1:1000; Jackson Immunoresearch, West Grove, PA), or Alexa Fluor 555-conjugated goat anti-mouse (1:1000; Life Technologies) and Alexa Fluor 488-conjugated goat anti-rabbit (1:1000; Jackson Immunoresearch) secondary antibodies were applied for a period of 1 hr at room temperature. The intensity of staining was measured in ImageJ (Schneider *et al.* 2012) after manual background subtraction using a nearby region off the process as described by Chen and Chalfie (2015). The average and SEM of the response of at least three biological replicates were reported and used for statistical analyses. Each biological replicate included 15−25 animals.

### Statistical analysis

Mann-Whitney *U*-test was used to evaluate single molecule mRNA FISH data. All other results were evaluated using Student’s *t*-test. All *P* values reported are after Bonferroni correction. Throughout the paper, we used “N” to indicate the number of biological replicates, each containing multiple animals, and “n” to indicate the number of individual animals.

## Results

### Feeding RNAi screen for genes affecting touch sensitivity

A total of 916 bacterial strains from the Ahringer RNAi library produced lethality or paralysis (Fraser *et al.* 2000; Kamath *et al.* 2003), but only 849 strains were available for the screen (the other bacterial strains were not viable). Five of these strains carried constructs targeting focal adhesion genes (*pat-2*, *pat-4*, *pat-6*, *unc-97*, and *unc-112*). These genes, which are known to affect touch sensitivity (Hobert *et al.* 1999; Calixto *et al.* 2010; Chen and Chalfie 2014), were included in our screen as blind positive controls.

To test whether other genes whose loss normally prevents testing of touch sensitivity (because of lethality or paralysis) affected mechanosensation, we performed neuronally enhanced RNAi in strain TU3595 (Calixto *et al.* 2010) and tested anterior touch sensitivity. TU3595 carries an *unc-119p*::*sid-1* construct that expresses *sid-1(+)*, a gene needed for the transport of dsRNA into cells (Winston *et al.* 2002), mostly in neurons in a *sid-1him-5*; *lin-15b* background. These animals showed enhanced neuronal RNAi phenotype and diminished RNAi phenotype in other tissues. RNAi to 61 genes, including all five positive controls, reduced touch sensitivity (Table 1). Twelve of the newly identified genes are involved in housekeeping functions such as general transcription and translation and are likely to cause general deficiencies in the TRNs. The remaining 44 newly identified genes are involved in transcriptional regulation, protein degradation, cellular adhesion, cytoskeletal structure and function, endo/exocytosis, mitochondrial function, calcium signaling, and other signaling cascades. One additional gene, *tba-1*/α-tubulin, was not included in the results because the RNAi construct targeting *tba-1* also targets a second α-tubulin, *mec-12*, which is needed for TRN mechanosensation (Fukushige *et al.* 1999).

**Table 1:** Genes that affect touch sensitivity

Gene Name	Description	Confirmed by Mutation
Transcription and translation related		
* Y47H9C.7*	EIF2B β subnit homolog	
* TAF-5*	TAF (TBP-associated transcription factor) family	
* taf-9*	TAF (TBP-associated transcription factor) family	
* nars-1*	Asparaginyl-tRNA synthetase	
* mog-5*	DEAH RNA helicase orthologous to PRP22 proteins.	Yes
* hars-1*	Aistidyl-tRNA synthetase (HisRS)	
* F54D5.11*	TFIIE β subunit	
* xrn-2*	5′->3′ exonuclease	
* C48E7.2*	RNAPol IIIC homolog	
* D2085.3*	EIF2B ε subunit	
* F55F8.3*	WD40-repeat-containing subunit of the 18S rRNA processing complex	
*F19F10.9*	Human SART1 homolog, U4/U6.U5 tri-snRNP−associated protein 1	
Transcription and splicing control		
* dmd-5*	Doublesex/mab-3 like	
* mfap-1*	Microfibrillar-associated protein homolog, controls alternative splicing	
Protein degradation		
* pas-4*	Proteasome α-type seven subunit of the core 20S proteasome subcomplex	
* rpn-1*	Non-ATPase subunit of proteasome 19S regulatory subcomplex	
Calcium signaling		
* mca-3*	Plasma membrane Ca^2+^ ATPase	Yes
* cal-2*	Calmodulin homolog	
* unc-43*	CaMKII	Yes
Adhesion/focal adhesion complex		
* pxl-1*	Paxillin 1	
* pat-2**^a^*	α-integrin subunit	Yes
* pat-4**^a^*	Integrin-linked kinase	Yes
* pat-6**^a^*	α-parvin (Actopaxin)	Yes
* unc-112**^a^*	Orthologous to human mitogen-inducible gene-2	Yes
* unc-97**^a^*	LIM domain-containing protein of the PINCH family	Yes
* lam-2*	Laminin γ subunit	
* hmr-1*	Classical cadherin	
Cytoskeleton and cell division		
* ifa-3*	Essential intermediate filament protein	
* pfd-3*	Putative prefoldin, orthologous to human VBP1 that is required for α-tubulin synthesis	
* tag-170/txdc-9*	Thioredoxin domain-containing protein orthologous to human TXNDC9	Yes
* cdk-1*	Cyclin-dependent kinase, orthologous to CDC28 from *S. cerevisiae*	Yes
* fzy-1*	An ortholog of *S. cerevisiae* Cdc20, predicted to regulate metaphase-anaphase transition	Yes
* knl-1*	Novel acidic protein, kinetochore component	
* myo-3*	Myosin heavy chain A	
Endo/exocytosis and synaptic functions		
* tom-1*	Tomosyn ortholog, binds SNAP25 (RIC-4)	Yes
* unc-11*	Clathrin-adaptor protein AP180	Yes
* cab-1*	Novel protein with a C-terminal motif weakly homologous to NPDC-1	Yes
Mitochondria		
* T20H4.5*	23-kDa subunit of mitochondrial complex I	
* Y37D8A.18*	Mitochondrial ribosomal protein, small	
* F43E2.7*	Mitochondrial carrier nomolog	
Signaling pathways		
* ptc-1*	Ortholog of *Drosophila* PATCHED (PTC) and human PTCH	Yes
* ptc-3*	Ortholog of *Drosophila* PATCHED (PTC) and human PTCH	
* goa-1*	Heterotrimeric G protein α subunit Go (Go/Gi class)	Yes
* kin-18*	TAO kinase	Yes
* let-502*	Rho-binding Ser/Thr kinase orthologous to human myotonic dystrophy kinase (DM kinase)	Yes
* let-92*	Catalytic subunit of protein phosphatase 2A (PP2A)	Yes
* F47F2.1*	PKA homolog	
* R03E1.2*	Renin receptor homolog	
Others		
* nsf-1*	NSF (*N*-ethylmaleimide sensitive secretion factor) homolog, may be required for ER to Golgi transport	
* gfi-2*	GEI-4 (four) interacting protein	
* glf-1*	UDP-galactopyranose mutase	
* K12H4.4*	Signal peptidase complex subunit	
* pnk-1*	Pantothenate kinase	Yes
* saps-1*	SAPS (phosphatase-associated) domain protein	
* vha-5*	Subunit a of the membrane-bound (V0) domain of vacuolar proton-translocating ATPase (V-ATPase)	Yes
* crn-1*	Cell death−related 5′-3′ exonuclease, homologous to mammalian flap endonuclease 1 (FEN1)	
* cgt-3*	Ceramide glucosyltransferase, required for glycosphingolipid production	Yes
* sqv-3*	β-(1,4)-galactosyltransferase, required for cytokinesis	Yes
* T19B10.2*	Nematode-specific protein	
* F26G1.2*	Nematode-specific protein	
C30B5.6	Unknown protein	

Gene names and their inferred homologies are listed. The genes were grouped according to their putative functions and/or the functions of their orthologs. tRNA, transfer RNA; ER, endoplasmic reticulum; SAPS, SIT4 phosphatase–associated protein.

a Five positive controls previously shown to affect touch sensitivity (Chen and Chalfie 2014).

**Figure 1:**
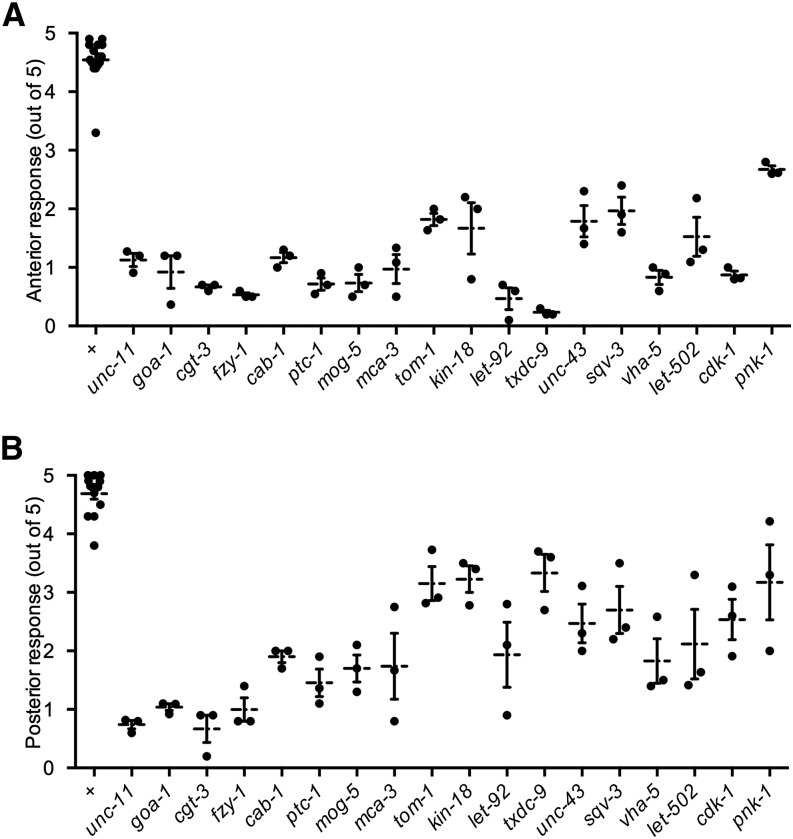
The anterior (A) and posterior (B) responses to five touches of the indicated animals. Individual data points mark mean response from a plate of animals. Mean and SEM of individual data points are also marked. N = 3 for all mutants and N = 14 for wild type. For all values, *P* < 0.05 compared with wild-type after Bonferroni correction except for *pnk-1* posterior response.

We tested the touch sensitivity of animals carrying loss-of-function alleles for 18 of the 61 genes. These strains either carried partial loss-of-function alleles or null alleles that were maternally rescued and, thus, produced young larvae whose touch sensitivity could be tested. All mutants tested produced anterior touch insensitivity (Figure 1A, *P* < 0.05 after Bonferroni correction), indicating that our screen had a low false-positive rate. Most of the mutants also showed defective posterior touch response to varying degrees (Figure 1B; *pnk-1* was not significantly different from wild type after Bonferroni correction).

**Figure 2:**
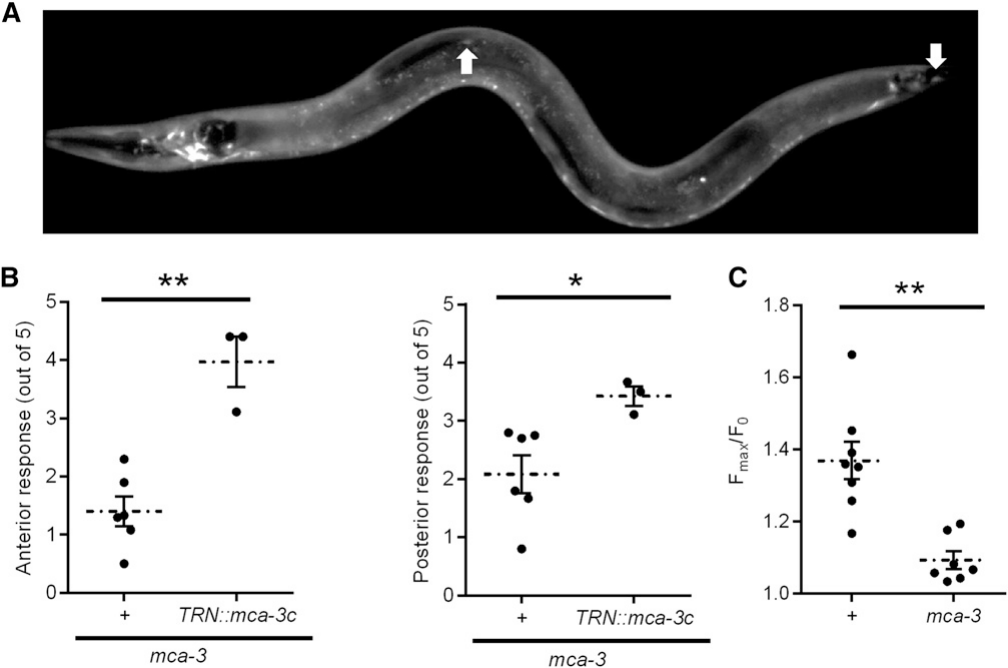
mca-3 is required for touch sensitivity. (A) *mca-3p*::*gfp* is expressed in the TRNs. The white arrows mark the ALM and PLM cells. (B) Anterior (left) and posterior (right) touch sensitivity of *mca-3* animals with or without *mec-3p*::*mca-3(+)* (*TRN*::*mca-3c*). Individual data points mark mean response from a plate of animals. Means and SEMs of the individual data points are also shown. *P < 0.05, and **P < 0.001. N ≥ 3. (C) Maximum calcium response elicited by touch in wild-type and *mca-3* animals, as detected by relative changes in GCaMP3 fluorescence. Individual data points as well as the means and SEMs are shown. n ≥ 7. **P < 0.001.

We did additional tests with one of the confirmed genes, *mca-3*. *mca-3* encodes a plasma membrane Ca^2+^ ATPase that maintains intracellular Ca^2+^ level by extruding cytosolic Ca^2+^ (Bednarek *et al.* 2007). Consistent with a previous report (Bednarek *et al.* 2007), *mca-3* is expressed throughout the body, including in the TRNs, as shown by *mca-3* promoter green fluorescent protein fusion (Figure 2A). A partial loss-of-function allele of *mca-3(ar492)* was viable and caused a partial reduction of touch sensitivity (Figure 2B). Expressing wild-type *mca-3* driven by a *mec-3* promoter restored the touch sensitivity, suggesting that *mca-3* acts cell-autonomously in the TRNs. The loss of touch sensitivity in *mca-3* mutant is further confirmed by reduced saturated calcium response elicited by touch (Figure 2C). These results indicate that *mca-3* affects touch sensitivity.

**Figure 3:**
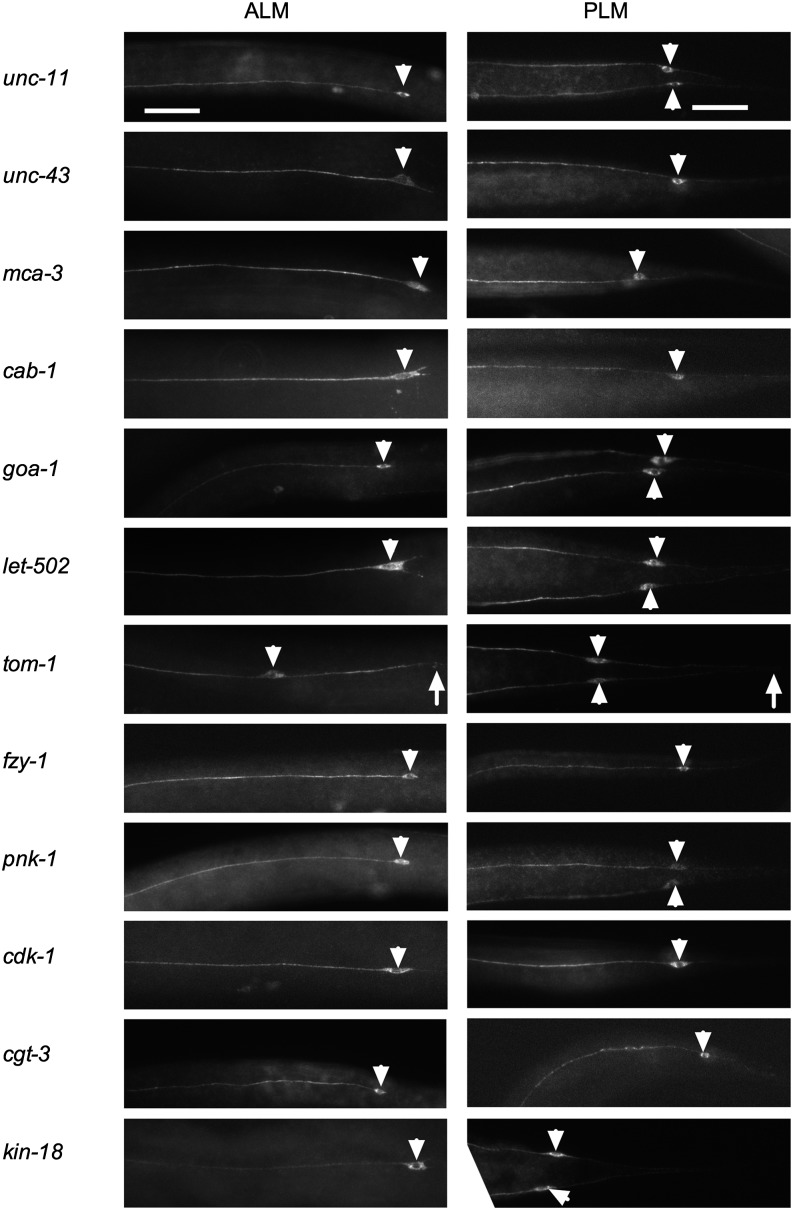
TRN morphology in touch-defective mutants. Representative images of MEC-18 staining in ALM and PLM processes of the indicated mutants. White arrowheads denote TRN cell bodies. One or two PLM cell bodies can be seen in each image depending on the focal plane. White arrows denote the end of posterior processes in *tom-1* mutants. Scale bar = 10 µm. TRN, touch receptor neuron.

To look for morphologic defects in the TRNs, we stained animals carrying mutations in 13 of the 18 genes with an antibody against MEC-18 (Figure 3; we were unable to characterize MEC-18 expression in *ptc-1*, *mog-5*, *let-92*, *sqv-3*, and *vha-5* mutants either because we were unable to identify homozygous mutants after fixation or because the homozygotes died too young). MEC-18 is expressed exclusively in the TRNs; except for the nucleus, MEC-18 is found throughout the TRNs. Eleven of the strains had grossly normal anterior (ALM) and posterior (PLM) TRNs, demonstrating that these genes are not needed for process outgrowth and that the cells have been generated.

Animals carrying a mutation in the two remaining genes, *tom-1* and *tag-170* (described below) had defective TRNs. Both the ALM and PLM neurons in *tom-1* mutants had longer posterior processes. We do not know whether this phenotype is related to the touch insensitivity seen in these animals, especially because *tom-1* is known to be involved in synaptic vesicle exocytosis (Gracheva *et al.* 2006).

### txdc-9 is preferentially required for touch sensitivity in the anterior TRNs

**Figure 4:**
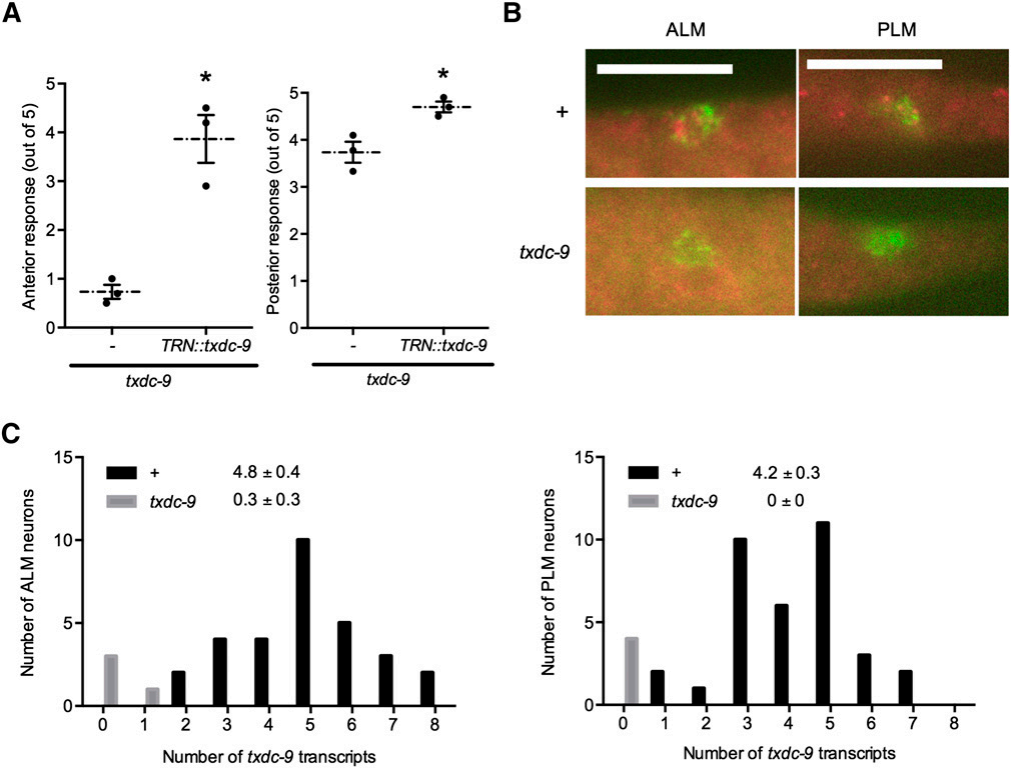
TXDC-9 is needed cell-autonomously for TRN functions. (A) Anterior (left) and posterior (right) responses to five touches of *txdc-9(ok776)* animals with or without *mec-3p*::*txdc-9(+)* [*TRN*::*txdc-9*]. Individual data points and the mean and SEMs are shown. N = 3 for all strains, *P < 0.005 for anterior touch and *P* < 0.05 for posterior touch. (B) Representative pictures of single molecule mRNA fluorescence *in situ* hybridization against *txdc-9* in the ALM and PLM neurons in wild-type and *txdc-9* animals. Scale bar = 5 µm. (C) Quantifications of the number of *txdc-9* transcripts in ALM and PLM neurons of wild-type (+) and *txdc-9* animals. *P < 0.0001 between wild type and *txdc-9*. TRN, touch receptor neuron.

*tag-170* encodes a conserved thioredoxin domain-containing protein homologous to TXNDC9 in humans, which is involved in microtubule growth and organization (Ogawa *et al.* 2004; Srayko *et al.* 2005). Because the gene name *tag* stands for *t*emporarily *a*ssigned *g*ene, we have renamed this genes *txdc-9* for *t*hioredo*x*in *d*omain-*c*ontaining protein 9. Because the specialized 15-protofilament microtubules unique to the TRNs are required for normal TRN function (Bounoutas *et al.* 2009, 2011), we studied this gene further. Because animals homozygous for the deletion mutation *txdc-9(ok776)* arrested at various larval stages (presumably because of maternal rescue), we compared maternally rescued *txdc-9* animals from a balanced heterozygous strain (VC556) and their heterozygous siblings. Some of the *txdc-9(ok776)* showed movement defects or were paralyzed. The maternally rescued *txdc-9* animals were insensitive to anterior touch, but retained partial posterior touch sensitivity (Figure 4A).

Neither a 400-bp nor a 2-kb promoter region upstream of *txdc-9* fused with *gfp* drove visible transgene expression (data not shown), but single-molecule mRNA fluorescence *in situ* hybridization (Topalidou *et al.* 2011) detected abundant *txdc-9* transcripts throughout the animal, including in the ALM and PLM neurons (Figure 4, B and C). No transcripts were detected in *txdc-9* homozygous mutants (Figure 4, B and C), indicating that our probe was specific for *txdc-9*. Because ALM and PLM neurons express *txdc-9* at similar levels, the difference in anterior and posterior touch sensitivity suggests that *txdc-9* is more critical to anterior touch sensitivity. Wild-type copies of *txdc-9* expressed under a *mec-3* promoter, which drives expression in all six TRNs and two additional pairs of mechanosensory neurons (Way and Chalfie 1989), restored anterior touch sensitivity in *txdc-9* animals and slightly rescued posterior touch sensitivity (Figure 4A), indicating that TXDC-9 acts cell-autonomously in the TRNs for optimal touch sensitivity.

### TXDC-9 is needed for microtubule function in the TRNs

**Figure 5:**
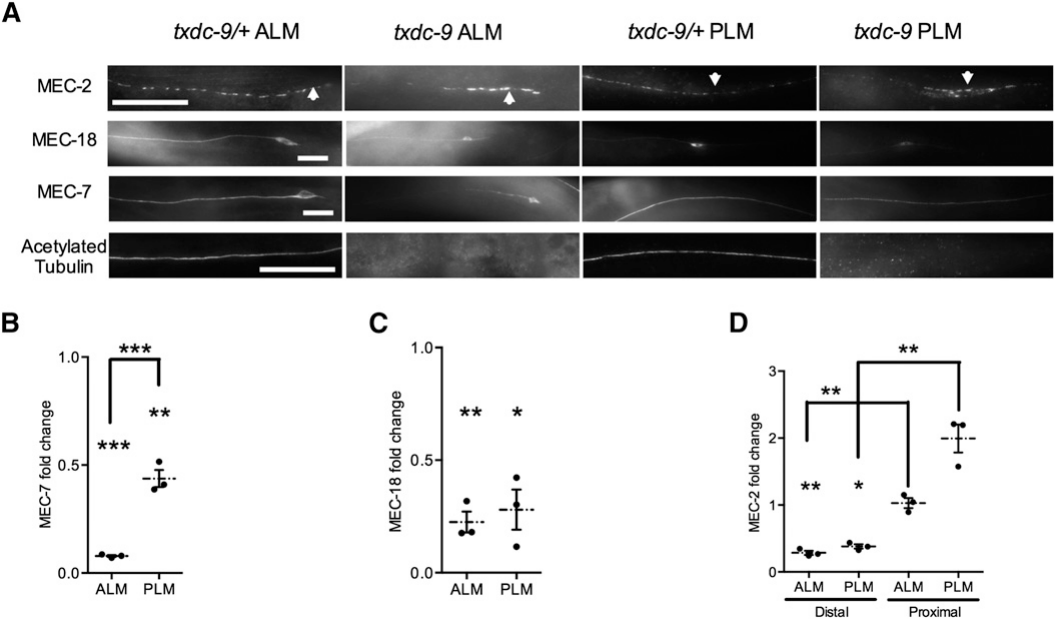
TXDC-9 differentially affects microtubule organization in ALM and PLM neurons. (A) Representative images of MEC-2, MEC-18, MEC-7, and acetylated tubulin staining in ALM and PLM processes of *txdc-9* and *txdc-9*/+ animals. Scale bar = 10 µm. (B−D) Quantifications of the fold change of (B) MEC-7, (C) MEC-18, and (D) MEC-2 comparing *txdc-9* to *txdc-9/+* animals. Individual data points, means, and SEMs are shown. *P < 0.05, **P < 0.01, and ***P < 0.001 comparing each set of data to the theoretical value of 1 or comparing two indicated sets of data. N = 3 for all data.

Consistent with a role of TXDC-9 in microtubule formation, *txdc-9* animals had reduced levels of MEC-7 β-tubulin (Figure 5, A and B) and no acetylated α-tubulin in the ALM and PLM processes [Figure 5A; fluorescence was not quantified for acetylated tubulin because staining was consistently seen only in heterozygotes (all 39 ALM neurons and 31 PLM neurons) but not in any homozygous mutant animals (all 23 ALM neurons and 22 PLM neurons)]. Although tubulin acetylation was eliminated in both ALM and PLM neurons, the reduction in MEC-7 was nearly sixfold greater in the ALM neurons than in the PLM neurons (6% of wild-type intensity in ALM neurons *vs.* 44% of wild-type intensity in PLM neurons, *P* < 0.0008; N = 3). These results indicate that TXDC-9 is needed for tubulin acetylation in both the ALM and PLM neurons, but it may be more critical for microtubules in the ALM neurons.

Disruption of microtubules in the TRNs also reduces expression of proteins essential for touch sensitivity, including MEC-2 and MEC-18 (Bounoutas *et al.* 2009, 2011). In *txdc-9*−deficient animals, the ALM and PLM processes also exhibited diminished levels of MEC-18 (Figure 5, A and C). MEC-2 levels were reduced in the distal processes but remained relatively unchanged or increased in the proximal processes (Figure 5, A and D; *P* < 0.01 comparing the proximal section to the distal section of both ALM and PLM neurons), suggesting defects in the transport of MEC-2 along the process. Similar defects were seen for both MEC-2 and MEC-18 in the ALM and PLM neurons, suggesting that these changes are unlikely to account for the differential touch sensitivity in *txdc-9* mutants. The reduced expression and transport defects in MEC-2 and MEC-18 distribution are consistent with defective microtubule organizations in these cells (Bounoutas *et al.* 2009, 2011).

## Discussion

We have systematically screened genes that cause nonviable phenotypes when mutated using tissue specific RNAi and have identified 61 genes that potentially affect mechanosensation in the TRNs (including five positive controls), out of 849 genes screened (Table 1). This ratio (61/849 = 7%) is much greater than the ratio of genes needed for touch sensitivity as identified by forward genetic screens (15/20,517 = 0.1%). Animals carrying mutations in many of these genes had TRNs with normal morphology (Figure 3), suggesting that reduction in the activities of these genes did not reduce touch sensitivity by affecting the general cellular functions or the development of the TRNs. We have estimated the false positive rate to be low (~2 of 61), but this estimation only included randomly occurring false-positive results. The actual false-positive rate may be greater because of possible off-target effects from the RNAi constructs. Nevertheless, the actual false-positive rate is probably still very low, given that all mutants tested were confirmed to be touch insensitive. Some of the candidates identified either form protein complexes with additional proteins (such as TAF-5 and IFA-3) or function similarly with other genes that are likely to be essential for viability (such as *nars-1* and *hars-1*). These additional genes were not identified in our screen, perhaps because they act redundantly, the RNAi was ineffective, or the genes were not essential for survival and thus were not screened.

In addition to genes needed for mechanosensation, we expected that this screen would also uncover genes needed for general cell functions. Indeed, at least 12 of the candidate genes are involved in cellular housekeeping functions, including general transcription factors, RNA polymerase subunits, and tRNA synthetases. One of these genes, *mog-5*, was confirmed using a maternally rescued null allele. Additional genes may also be needed for cellular housekeeping but were not discovered in our screen, probably due to redundancy.

A total of 17 other nonhousekeeping genes were confirmed using maternally rescued null alleles or partial loss-of-function alleles. The nonhousekeeping genes, however, do not necessarily function specifically in mechanosensation. Candidate genes in several groups, including protein degradation, calcium signaling, cytoskeleton, mitochondria, and synaptic functions, are likely to affect neuronal functions in general. Some of these genes, nevertheless, may have specific roles in the TRNs.

One of the candidate genes, *txdc-9*, is needed for microtubule function and is conserved from yeast to humans (Ogawa *et al.* 2004). Although we have identified a function of TXDC-9 in mechanosensation, TXDC-9 generally is required for normal spindle formation during cell division (Ogawa *et al.* 2004). The mammalian homolog of *txdc-9*, *TXNDC9*, modulates tubulin folding in complex with the chaperone CCT, maintains β-tubulin levels, and promotes cytoskeletal remodeling (Stirling *et al.* 2006; Hayes *et al.* 2011). The *C. elegans cct* genes also are needed for optimal touch sensitivity and microtubule acetylation (Keller 2011). Consistent with a general role of TXDC-9 in microtubule dynamics, animals homozygous for the *txdc-9* mutation were defective in movement, a phenotype also seen when microtubules are disrupted by benomyl and other microtubule drugs (Chalfie and Thomson 1982).

We find that *txdc-9* is needed for tubulin acetylation and the function of the specialized microtubules in the TRNs, which are needed for normal mechanotransduction (Chalfie and Sulston 1981; Chalfie and Thomson 1982; Bounoutas *et al.* 2009). These specialized microtubules are heavily acetylated by MEC-17, an α-tubulin acetyltransferase (Akella *et al.* 2010), but this acetylation is not needed for mechanotransduction (Topalidou *et al.* 2012). Thus, the effect of *txdc-9* loss on touch sensitivity and MEC-7/β-tubulin levels is likely to be independent of its effect on tubulin acetylation in both ALM and PLM neurons. Other defects in the TRNs, including the changes in the expression and distribution of MEC-2 and MEC-18, also are consistent with a more general role of TXDC-9 in microtubule function and organization. Because these defects were stronger in the ALM neurons than in the PLM neurons, they reveal TRN subtype differences in microtubule activity.

Genes involved in classical signaling cascades may affect TRN functions more specifically. A previous RNAi screen for genes involved in integrin signaling found that the insulin-like pathway is involved in gain control in the TRNs (Chen and Chalfie 2014). Hilliard and Bargmann (2006) showed that Wnt signaling establishes the correct morphology of the TRN processes. We have identified and confirmed genes in several additional classical signaling pathways, including Hedgehog signaling pathway (*ptc-1*), G proteins (*goa-1*), and Rho GTPase (*let-502*), suggesting that these signaling pathways may also affect the function or the development of the TRNs.

Genes involved in focal adhesion complexes have been shown previously to be involved in TRN functions (Calixto *et al.* 2010; Chen and Chalfie 2014). In this screen, we identified two additional adhesion molecules needed for touch sensitivity. *pxl-1*, encoding a Paxillin, likely functions with the other focal adhesion proteins (Warner *et al.* 2011) in regulating touch sensitivity. *hmr-1* encodes a classical cadherin that functions independently of the focal adhesion complexes. This result suggests that the cadherin complexes also may contribute to mechanosensation in the TRNs.

In summary, our results suggest that screens using neuronally enhanced feeding RNAi complement traditional forward mutagenesis screens in identifying genes needed for neuronal functions. The interpretation of the candidates from such screens, however, requires confirmation and further analysis using null alleles. In addition, mosaic analysis can be used to overcome lethality by testing animals lacking the essential genes specifically in the TRNs.
